# Not God's Fault, but Ours

**Published:** 1909-11-13

**Authors:** 


### The Hospital

A Journal of

The Medical Sciences and Hospital Administration.

New Series. No. 142, Vol. VI. [No. 1214, Vol. XLVII.] SATURDAY, NOVEMBER 13, 1909.

### "Not God's Fault, but Ours."

As the foundation of material strength is finance, so the foundation of physical well-being is health. Hence Society owes it to the weakest members of the race that nothing shall be left undone which can promote and maintain the health of each individual, however humble his origin. The Bishop of Birmingham, Dr. Gore, made a new departure in his sermon on Hospital Sunday at St. Laurence's Church, Birmingham, on October 31. He dealt with the sins of society in regard to its neglect of the poor. He showed in an eloquent discourse many directions in which the well-being and life of the English people of the poorer class are neglected and often ruined, not by God's fault but by Society's neglect of a plain duty. Bishop Gore has the merit of being a preacher of directness, and we hope that his sermon may be printed and circulated throughout the country.

Hospital Sunday is a national institution which has done immense good by uniting all the sects on one common platform in the cause of suffering humanity. It has done much to maintain and preserve the voluntary system of hospital support, under which system British hospitals to-day have become the most efficient institutions in the world. May the Bishop of Birmingham's example be followed by all the Bishops and teachers of every denomination, and may they use their influence in the future to make Hospital Sunday a day on which every preacher, led by the Bishop of the diocese, will direct his efforts to a moral stocktaking of the social condition of the people in this country. The clergy and ministers have the best opportunities for knowing how and where the neglect of society injures the poor. Let the clergy approach their Bishop, or the head of their denomination, a fortnight before Hospital Sunday, and by sending him particulars of the pressing needs and avoidable sufferings of their people through no fault of their own, put him in a position to deliver a powerful address each year on this day. Such a crusade against the avoidable evils which tend to destroy the health of the people must result in an awakening of the moral conscience of all classes, and the people will thus come to recognise that many things affecting the life and health of the poor which are now acquiesced in or tolerated must be speedily remedied and removed. Of course, the main object of the Hospital Sunday Fund movement has been to raise money for the hospitals. We make bold to say, however, that the general adoption of this suggestion would arouse such an interest in the Hospital Sunday services as to ensure that the contributions upon that day, everywhere, would be greatly increased, whilst the social condition of many classes of the people would be made infinitely better than it is to-day.

Again, the Bishop of Birmingham's sermon should cause the thoughtful to realise that every independent person in these islands has a responsibility for each hospital patient who has arrived at the stage where disease has ceased and health has to be restored. The modern voluntary hospital is a place where the maximum of good is effected by the admission of acute cases and their treatment, under conditions which have reduced the number of days' residence in hospital to nearly one-half of what they were thirty years ago. Formerly some eight or nine patients passed through each bed in the course of twelve months; now it has become possible to treat double that number of patients, and so to increase immeasurably the value and importance of voluntary hospital treatment. Even the present shortened days of residence might be materially reduced if ordinary people would interest themselves in hospital patients, and form a committee in each urban and rural centre where hospitals exist, with the object of providing that convalescent care shall be made available for every patient who would benefit by removal from the hospital into the country at the earliest possible moment. Manchester has a Surgical Aid Society which provides for patients who require surgical instruments and appliances. That Society, and all similar societies, might add to their usefulness by lending a helping hand to every hospital patient who arrives at the stage where convalescence begins.

Bishop Gore insists with great force and truth that many people die under existing conditions, not because it pleases God, but because society has condemned them to sweated wages and to live lives that are not lives, because they are starved and maimed lives. Thus society misuses and maltreats such persons, and does things which are not in accord with God's will, for they are flatly contradictory to it. It is God's will that such evils should cease, and that we should be stirred up to rebellion against them. "It is not God's fault, but ours, that so many babies die. Bad feeding of babies, bad housing, the bringing up of children so that they learn no useful trade, but drift into casual labour or a sweated industry, the crowding of people together so that they cannot be healthy—these are all evils which God would have us alter, and they can be altered if people will agree to postpone their selfish interests to the interests of their brethren." The ministry of the church ought to be a ministry to resist disease, and the aim of leaders of public opinion ought to be a ministry of personal service to transform sickness into health. Why should not every mayor and every chairman of a county council devote some time this winter to stirring up the people in their municipalities and counties to realise their responsibility for the long-permitted and preventable evils which press upon the helpless poor, evils which are not God's fault, but ours, as the Bishop of Birmingham wisely insists. We hope that something may be done during the ensuing months throughout the country to tackle this important aspect of the responsibility of living. We venture to say that every one of the leaders of opinion to whom we have referred who puts himself at the head of a movement to rectify these things will be made immeasurably happier by such action than he has ever been before during his whole life. We hope, too, that Hospital Sunday during 1910 in every part of the United Kingdom will be made a day of social stocktaking in the best interests of all classes of society, as well as of the sick and suffering poor.

